# The microbiota, antibiotics and breast cancer

**DOI:** 10.2217/bmt-2019-0015

**Published:** 2019-10-18

**Authors:** Alastair M McKee, Lindsay J Hall, Stephen D Robinson

**Affiliations:** 1 Gut Microbes & Health, Quadram Institute Bioscience, Norwich Research Park, Norwich, UK; 2 School of Biological Sciences, University of East Anglia, Norwich Research Park, Norwich, UK

**Keywords:** antibiotics, breast cancer, microbiota

Breast cancer is responsible for the highest occurrence and mortality rates among the global female population, with one in eight British women diagnosed with the disease in 2019 [1]. While the 5-year survival of a patient presenting with stage 1 or 2 cancer is over 80%, patients presenting with stage 4 disease have only a 15%, 5-year survival rate [2].

Breast cancer is a heterogeneous disease classed into four major subtypes according to the presence or absence of the hormone receptors for estrogen and progesterone as well as HER2. Histological characterization of receptor status has paved the way for personalized treatments, with gene expression analysis further refining this profiling. Despite this attempt at a more ‘personalized’ treatment approach, most women receive surgery. This can either be a total mastectomy or breast conserving surgery to remove the tumor while maintaining healthy breast tissue [1,3]. For many women, this inevitably means receiving systemic antibiotic treatment before and/or after surgery.

While antibiotics represent a critical treatment for bacterial infection, they do not discriminate between pathogenic and more beneficial microbes, such as those that reside in the gut, in other words, the microbiota. The gut microbiota represents a diverse and dense microbial ecosystem that plays a key role in promoting health under normal homeostatic conditions including, metabolizing dietary components in our food, infection resistance and developing and priming the immune system [4,5]. Given its key role in regulating immune processes, including both innate and adaptive responses at mucosal and system levels, it is not surprising that the gut microbiota has recently been described as playing a role in programming anticancer responses.

It is increasingly clear that the composition and diversity of the gut microbiota impacts tumor immune responses [6–8], but detailed mechanistic understanding of these responses is lacking. Moreover, current cancer microbiota studies are largely focused either on cancers local to the gut or on highly immunogenic primary cancers (lung, kidney, bladder, melanoma); cancers known to respond well to immune-based therapies. Until recently, there has been little focus on microbiota and cancers that, historically, respond less favorably to immune-check point therapies, such as breast cancer. Even less analysis has been done on investigating the relation between gut microbiota and metastasis.

Recently, a number of studies have started to address these gaps in our knowledge using mouse models of breast cancer [9,10]. Use of preclinical models allows for a more detailed mechanistic probing of potential underlying effects of microbiota modulation than can be performed in breast cancer patients and allows ‘targets’ to be identified that can be followed in next stage clinical studies. These studies identified that a ‘balanced’ gut microbiota is essential for programming host responses, which directly impacts tumor outcomes. As highlighted above, breast cancer patients are commonly exposed to antibiotic treatment and both studies tested the hypothesis that antibiotic-induced perturbations of the gut microbiota may impact breast cancer progression. Notably, although different mouse models were used, findings indicated that antibiotic exposure significantly accelerated progression of the cancer. However, the mechanisms behind how antibiotic-induced microbiota disturbances accelerate progression in these two studies was different (immune vs metabolic), which may link to the differing treatment schedules, and thus impact on the gut microbiome. Nonetheless, the findings of enhanced primary tumor progression in multiple mouse models, and with multiple antibiotic regimes, suggests these principles may apply in human breast cancer and across the spectrum of the disease. Thus, an important message of these studies is that we need to think carefully about how and when we use antibiotics in breast (and other) cancer patients.

Alongside the microbiota-modulating effects of antibiotics, there is another serious threat to consider in the clinic. The rise of antibiotic-resistant pathogens is of such concern, it has been highlighted at the top and global levels in the medical profession as well as within governments. Antimicrobial resistance is already killing 700,000 people per year worldwide and this figure is expected to grow exponentially over the next 30 years [11]. Indeed, routine medical procedures, like surgery for tumor removal in breast cancer patients, may become too ‘dangerous’ to undertake if the global threat of antimicrobial resistance is not counteracted. Furthermore, there is already some evidence to suggest that antibiotic use may not be beneficial to all breast cancer patients [12]. It is therefore paramount that we begin to carefully consider whether using antibiotics will be effective in patients. Where their use is unavoidable, further work needs to address crucial questions with respect to antibiotic treatment choice and/or ways to counteract their negative effects.

There are several important considerations to be addressed to provide clear guidance in the clinic for healthcare professionals to enhance positive outcomes in patients. These include:

- We need to understand what a balanced microbiota looks like when it comes to dictating breast cancer outcomes. This will only come with further fundamental research into what the microbiota looks like in both mouse models and breast cancer patients. Importantly, it is essential we understand not only ‘who’ is present (e.g. through metagenomic sequencing), but also ‘what’ they can do (e.g. through transcriptional and/or metabolic profiling).
- It is clear there are no firm guidelines on what antibiotics should be prescribed prophylactically to prevent surgical site infections. Thus, we need a better understanding of how different antibiotic regiments might influence breast cancer progression (via gut microbiota-induced disturbances) with preclinical models a natural choice for teasing these potentially complex effects.
- We must also systematically interrogate and integrate the multiple cellular and molecular pathways that come together to govern the microbiota–breast cancer axis. Only by doing so will we understand how we might therapeutically intervene to realign the pathways after antibiotic-induced disturbances occur.

On a final note of caution, it is important to remember that what applies in breast cancer may not apply in other cancers. To date, different cancers seem to respond differently to microbiota manipulations. Like breast cancer, antibiotic-induced microbiota disturbances accelerate disease progression in melanoma [13], but have the opposite effect in pancreatic cancer [14]. Thus, examining the role of the microbiota in diverse cancers, using appropriate models and cancer patient samples, is a key therapeutic strategy. Indeed, these studies may provide an opportunity to beneficially modulate the gut microbiota after antibiotic disturbances such as through microbiota and/or dietary supplementation and treatment. Indeed, evidence is mounting to indicate that it is possible to manipulate gut bacterial communities in beneficial ways to improve the efficacy of some treatments. For example, mice harboring melanoma show improved responses to anti-PD-L1 immune checkpoint therapy when subjected to *Bifidobacterial* supplementation of their gut microbiota [7]. Strategies like these are yet to be tested on breast cancer, which historically has responded poorly to immune-based therapies.
